# Prevalence of High Bleeding Risk among Hospitalized Suspected NSTEMI Patients

**DOI:** 10.3390/jcm11051324

**Published:** 2022-02-28

**Authors:** Henri Kesti, Henna Mäkinen, Kalle Mattila, Samuli Jaakkola, Mikko Lintu, Pekka Porela

**Affiliations:** 1 Heart Centre, Turku University Hospital and University of Turku, 20521 Turku, Finland; samuli.jaakkola@tyks.fi (S.J.); pekka.porela@tyks.fi (P.P.); 2 Emergency Department, Turku University Hospital, 20521 Turku, Finland; henna.m.makinen@utu.fi (H.M.); kalle.matias.mattila@tyks.fi (K.M.); mikko.lintu@tyks.fi (M.L.); 3 Faculty of Medicine, University of Turku, 20014 Turku, Finland

**Keywords:** non-ST-segment elevation myocardial infarction, bleeding, high bleeding risk, Academic Research Consortium

## Abstract

In recent years, guidelines for the management of acute coronary syndromes (ACS) have placed more emphasis on identifying patients at high bleeding risk (HBR). We set out to investigate the prevalence of HBR patients according to the Academic Research Consortium for High Bleeding Risk (ARC–HBR) criteria in hospitalized patients with suspected non-ST-segment elevation myocardial infarction (NSTEMI). Consecutive patients were retrospectively enrolled between January and June 2019 from the emergency department (ED) of a tertiary hospital. The discharge diagnosis and baseline data were manually collected using electronic patient records and database searches. Patients with non-cardiac diagnoses were excluded. Overall, 212 patients were included in the study. A total of 146 (68.9%) patients were diagnosed with NSTEMI (Type 1), 47 (22.2%) with unstable angina pectoris (UAP) and 19 (9.0%) with “other.” HBR was detected in 47.6% (*n* = 101) of all patients. Common criteria for HBR among ACS patients were age (40.4%), chronic kidney disease (33.7%), and the use of oral anticoagulation medicines (20.2%). In conclusion, nearly half of the patients hospitalized for ACS fulfilled HBR criteria. According to contemporary guidelines, the management of HBR patients differs from that of non-HBR patients, and thus, a more comprehensive screening for HBR may be considered in clinical practice.

## 1. Introduction

The benefit of dual antiplatelet therapy (DAPT) in reducing further thrombotic events in acute coronary syndromes (ACS) after percutaneous coronary intervention (PCI) is well established by numerous studies [1,2,3,4,5]. However, it comes with a price of increased bleeding complications [2,5,6].

Identifying patients at high bleeding risk (HBR) in this group could be important in reducing bleeding complications associated with ACS management. Major bleeding has been shown to be an independent predictor for mortality, equaling the risk associated with ischemic complications [7,8,9,10,11]. Thus, the choice of treatment should be decided upon based on both the ischemic risk and the bleeding risk of the individual patient [11,12,13]. A recent study demonstrated that the clinical presentation of ACS per se predicts increased bleeding risk, as compared to chronic coronary syndrome, further emphasizing the importance of considering these two aspects during decision making [14].

In their most recent guideline for the management of non-ST-segment elevation acute coronary syndromes (NSTE–ACS) [13], the European Society of Cardiology (ESC) proposed the use of the Academic Research Consortium for High Bleeding Risk (ARC–HBR) criteria for the identification of HBR patients [15]. The consortium defined HBR as Bleeding Academic Research Consortium (BARC) Type 3 or 5 bleeding risk ≥ 4% or intracranial hemorrhage ≥ 1% at 1 year [16]. The criteria are separated into major and minor. Even a single major criterion induces the aforementioned bleeding rates, and a minor criterion induces lesser bleeding rates. Patients are at HBR if at least one major or two minor criteria are met.

Most of the data on bleeding events post-PCI comes from DAPT studies or PCI registries. Patients at HBR are often excluded from these studies. Furthermore, due to PCI being an invasive procedure, such registries are selective by nature. The information on the prevalence of HBR and bleeding complication rates among patients with NSTE–ACS is scarce. While there are tools for identifying HBR patients—such as predicting bleeding complications in patients undergoing stent implantation and subsequent dual antiplatelet therapy (PRECISE–DAPT) score [17]—these have not been properly utilized in daily clinical practice. The aim of this study was to investigate how many of the NSTE–ACS patients fulfilled ARC–HBR criteria for high bleeding risk in an unselected, non-register-based patient population.

## 2. Materials and Methods

The study population consisted of patients who were admitted to the emergency department (ED) due to “chest pain” between 1 January 2019 and 30 June 2019. Admission data was collected using the patient-information system Safir Spider, version 2.22.101.2461 (San Sai Solutions Oy, Turku, Finland), used by the ED at Turku University Hospital (Turku, Finland). If a patient had several visits, only the first one was registered. Only one NSTE–ACS event was reported per patient. We included all consecutive patients with suspected non-ST-segment elevation myocardial infarction (NSTEMI) (age ≥ 18 years) who were hospitalized. The primary discharge diagnosis was manually evaluated by the authors using electronic patient records and a database search conducted by Auria Clinical Informatics. The search identified a total of 2562 patients, 432 of whom were hospitalized, and after selecting those with NSTEMI and unstable angina pectoris (UAP) diagnosis codes, 212 patients were included in this study. Patients with non-cardiac diagnosis were excluded. The fourth universal definition of myocardial infarction (MI) was used to differentiate between Type 1 and Type 2 MIs [18]. In this study, patients with Type 2 MI were separated from the NSTE–ACS and NSTEMI groups.

Auria Clinical Informatics is an information service provider operating in connection with The Hospital District of Southwest Finland. The service was used to obtain information on dates of emergency department visits, relevant ICD-10 diagnosis codes and procedure codes (during the visits and within the following 60 days), relevant laboratory values during the hospitalization episode, and long-term diagnosis codes that were valid at the time of the visits.

ARC–HBR criteria were manually evaluated by the authors using the aforementioned methods. Relevant time periods were evaluated based on the date of hospitalization. Patients were defined as being at HBR if at least one major or two minor ARC–HBR criteria were met. Major and minor criteria were defined as follows: age ≥ 75 years (minor); use of oral anticoagulation (OAC) at discharge (major); severe or end-stage chronic kidney disease (CKD) with estimated glomerular filtration rate (eGFR (CKD-EPI formula)) < 30 mL/min (major); moderate CKD with eGFR ≥30, <60 mL/min (minor); hemoglobin < 11 g/dL at baseline (major); hemoglobin ≥ 11 g/dL, <13 g/dL at baseline for men and ≥11 g/dL, <12 g/dL at baseline for women (minor); spontaneous non-intracranial bleeding requiring hospitalization or transfusion in the past 6 months or at any time if recurrent (major); spontaneous non-intracranial bleeding requiring hospitalization or transfusion within the past 12 months not meeting the major criterion (minor); platelet count <100 × 10^9^/L at baseline (major); chronic bleeding diathesis (major); liver cirrhosis with portal hypertension (major); long-term use of oral non-steroidal anti-inflammatory drugs (NSAIDs) or steroids (minor); cancer diagnosis within 12 months (excluding non-melanoma skin cancer) and/or ongoing requirement for treatment (major); previous spontaneous intracranial hemorrhage (ICH) at any time, previous traumatic ICH within the past 12 months, ischemic stroke within the past 6 months, presence of brain arteriovenous malformation (major); any ischemic stroke at any time not meeting the major criterion (minor); nondeferrable major surgery on DAPT within 30 days after hospitalization (major); recent major surgery or major trauma within 30 days before hospitalization (major). For one patient, creatinine had not been recorded, resulting in missing eGFR. This patient was at HBR even without considering eGFR and was included in the study population.

Continuous variables are expressed as mean values with standard deviations, and categorical variables are presented as frequencies (percentages). Categorical variables were compared with Pearson’s chi-square or Fisher’s exact test, as appropriate. The comparison of two mean values for continuous variables was done with an independent samples *t*-test. Normality assumptions were verified using the Kolmogorov–Smirnov test, skewness, and kurtosis. Statistical analyses were performed using SPSS, version 27.0 (SPSS Inc., Chicago, IL, USA).

## 3. Results

The baseline characteristics and prevalence of HBR are shown in Table 1. The primary discharge diagnoses were as follows: 146 (68.9%) NSTEMI (Type 1), 47 (22.2%) UAP, 13 (6.1%) NSTEMI (Type 2), and 6 (2.8%) Takotsubo cardiomyopathy. HBR was detected in 47.6% (*n* = 101) of patients. In the NSTEMI group, 46.6% (*n* = 68), and in the UAP group 44.7% (*n* = 21), were at HBR.

Of the enrolled patients, 32.1% (*n* = 68) were female. The mean age was 71.7 (standard deviation (SD) ± 11.5) years. Mean age in the HBR group was 78.4 (SD ± 8.3) years vs. 65.6 (SD ± 10.6) years in the non-HBR group (*p* < 0.001). Additionally, 58.8% (*n* = 40) of females vs. 42.4% (*n* = 61) of males were at HBR (*p* = 0.025).

Of all study patients, 53.8% (*n* = 114) underwent PCI, 8.0% (*n* = 17) coronary artery bypass grafting (CABG), and 17.9% (*n* = 38) angiography without revascularization, while 20.3% (*n* = 43) were assigned to non-invasive management. Non-invasive management was more common in the HBR group than in the non-HBR group (74.4% vs. 25.6%, *p* < 0.001), whereas CABG was more common in the non-HBR group than in the HBR group (76.5% vs. 23.5%, *p* = 0.038). In the PCI group, 43.0% (*n* = 49) were at HBR vs. 57.0% (*n* = 65) who were non-HBR (*p* = 0.143). Among those who underwent angiography without PCI or CABG, 42.1% (*n* = 16) were at HBR vs. 57.9% (*n* = 22) who were non-HBR (*p* = 0.451).

The prevalence and distribution of different ARC–HBR criteria is shown in Table 2. In NSTE–ACS patients, the most common major criterion was use of OAC (20.2%, *n* = 39), including direct oral anticoagulants (*n* = 31) and vitamin K antagonists (*n* = 8). The most common minor and overall criterion was age ≥ 75 years (40.4%, *n* = 78). Other common minor criteria included moderate CKD (28.0%, *n* = 54), mild anemia (18.7%, *n* = 36), stroke minor (9.8%, *n* = 19), and long-term use of p.o. NSAIDs or steroids (7.3%, *n* = 14) (none were using NSAIDs). Other common major criteria included severe or end-stage CKD (5.7%, *n* = 11) and hemoglobin < 11 g/dL (6.2%, *n* = 12).

## 4. Discussion

Our study indicates that HBR is frequently encountered in this patient group, with almost half of the patients fulfilling ARC–HBR criteria. Notably, high age, use of OAC, moderate CKD, and mild anemia were common constituents of HBR criteria in these patients. Patients at HBR were older, more often female, and non-invasive management strategy was more common, as compared to non-HBR patients. Almost all the criteria were more prevalent in older patients.

The prevalence of HBR according to ARC–HBR criteria in studies of European and Asian populations has ranged from 30% to 50%, and the incidence of major bleeding events in the HBR groups of these studies has consistently been ≥4% at 1 year [14,19,20,21,22,23,24]. The highest major bleeding rates were reported in Korea. HBR was detected in 35.4% of Korean patients, and the incidence of major bleeding among this group at 1 year was 31.3% (BARC 3 to 5), even though some HBR factors—such as OAC use—were selected as exclusion criteria [22]. In a study concerning over 16,000 patients from Bern PCI registry, 34.7% were at HBR, and the incidence of major bleeding at 1 year was 7.9% (BARC 3 or 5) [19].

Gimbel and colleagues reported a bleeding incidence (PLATO major or minor) of 18% for clopidogrel versus 24% for ticagrelor users in patients aged ≥ 70 years presenting with NSTE–ACS [25,26]. In a sub-analysis of OAC users, bleeding rates were 20.9% and 33.5%, respectively [27]. While the latter are at HBR according to ARC–HBR criteria, the studies vastly excluded patients with increased bleeding risk [28].

In general, the majority of the data on bleeding events post PCI comes from clinical DAPT trials and PCI registries. Since PCI is an invasive procedure, the prevalence of HBR patients could be skewed to the lesser side when the data comes from said registries. In DAPT trials, HBR or some of its underlying constituents are often selected as exclusion criteria. Studies show that bleeding risk gets incrementally higher as risk factors, such as ARC–HBR, accumulate [19,20,21,22,23,24]. As a result, the prevalence of HBR and actual bleeding rates could be even higher than reported.

In our population, the most frequently encountered criteria were age, CKD, anemia, and use of OAC. Prior stroke and use of p.o. NSAIDs or steroids were moderately prevalent. Previous studies have reported similar results [14,19,20,21,22,23,24]. OAC-use and age seem to be even more prevalent in Finnish patients, but active malignancy is less frequently encountered. It is noteworthy that several of the minor ARC–HBR criteria in isolation predict a major bleeding incidence ≥ 4% at 1 year [19,20,21,22], particularly age, moderate CKD, and mild anemia, which are also the most prevalent ones.

For NSTE–ACS patients, the ESC guideline recommends that, after coronary stent implantation, DAPT consisting of acetylsalicylic acid (ASA) and a P2Y12 receptor inhibitor, should be continued for 12 months unless there are contraindications [1,2,5]. Discontinuation of a P2Y12 receptor inhibitor after three months should be considered in HBR patients [12,17]. According to Costa and colleagues, this also applies to patients with high ischemic risk. Longer-lasting DAPT was only beneficial when HBR was not present [29]. Alternatively, stopping ASA after 3 to 6 months should be considered, based on the balance between ischemic and bleeding risk [30,31,32]. The recommendation to cease aspirin is supported by recent reports investigating ticagrelor monotherapy after 3 months of DAPT [33,34]. Still, these recommendations are based on limited clinical data, and further study of antithrombotic medication in HBR patients is needed. Regarding the choice between clopidogrel and more potent inhibitors, the findings of Gimbel and colleagues suggest that clopidogrel is a better option compared to ticagrelor for ≥70 year-old HBR patients [25,27]. Choosing the right thienopyridine and suitable DAPT duration, and thus following current guidelines, requires identification of HBR patients.

Even shorter DAPT durations have recently been investigated. Valgimigli and colleagues concluded that, in HBR patients, 1 month of DAPT followed by 11 months of single antiplatelet therapy (SAPT) was non-inferior to at least 3 months of DAPT followed by SAPT regarding net adverse clinical events and resulted in lower incidence of major bleeding [35]. In the 1-month DAPT group, about 54% used clopidogrel monotherapy, and only about half the patients had an ACS. In a study enrolling exclusively ACS patients, however, 1 month of DAPT followed by 11 months of SAPT using clopidogrel failed to prove non-inferiority to 12 months of DAPT for net clinical benefit [36]. These studies were not focused on NSTE–ACS and had different patient demographics. The latter was focused on ACS but had 56% ST-segment elevation myocardial infarction patients, while the former had only about 12% and was focused on HBR patients. Different stent types were also used. It remains unclear whether 1-month DAPT is sufficient in NSTE–ACS.

Our study has some limitations. First, it was a retrospective study. Evaluation of the discharge diagnoses was done retrospectively, and thus, the investigators were reliant on the treating physicians. Second, it was a single-center study with a limited number of patients, which impairs the generalizability of the results. However, previous studies have reported similar prevalence rates.

In conclusion, HBR is frequently encountered in clinical practice, and more comprehensive screening is necessary for proper management of NSTE–ACS. The prevalence of HBR and bleeding rates should be investigated in a multi-center, prospective approach.

## Figures and Tables

**Table 1:** Baseline characteristics.

Variable	Total (*n* = 212)	HBR (*n* = 101)	Non-HBR (*n* = 111)	*p*-Value
Age	71.7 ± 11.5	78.4 ± 8.3	65.6 ± 10.6	<0.001
Sex				
Female	68	40 (58.8)	28 (41.2)	0.025
Male	144	61 (42.4)	83 (57.6)	0.025
Clinical presentation				
NSTEMI (Type 1)	146	68 (46.6)	78 (53.4)	0.644
NSTEMI (Type 2)	13	11 (84.6)	2 (15.4)	0.006
UAP	47	21 (44.7)	26 (55.3)	0.645
Takotsubo	6	1 (16.7)	5 (83.3)	0.215
Management				
Non-invasive	43	32 (74.4)	11 (25.6)	<0.001
Angiography without revascularization	38	16 (42.1)	22 (57.9)	0.451
PCI	114	49 (43.0)	65 (57.0)	0.143
CABG	17	4 (23.5)	13 (76.5)	0.038

Values are *n* (%) or mean ± standard deviation. *p*-values are between HBR group and non-HBR group. NSTEMI: non-ST-segment elevation myocardial infarction; UAP: unstable angina pectoris; Takotsubo: Takotsubo cardiomyopathy; PCI: percutaneous coronary intervention; CABG: coronary artery bypass grafting; HBR: high bleeding risk.

**Table 2:** Prevalence of ARC–HBR criteria.

Variable	Total (*n* = 212)	NSTE–ACS (*n* = 193)	NSTEMI (*n* = 146)	UAP (*n* = 47)	Age ≥ 75 (*n* = 88)	Age 65–74 (*n* = 71)	Age < 65 (*n* = 53)
OAC	42 (19.8)	39 (20.2)	26 (17.8)	13 (27.7)	30 (34.1)	10 (14.1)	2 (3.8)
Severe or end-stage CKD (eGFR < 30 mL/min)	14 (6.6)	11 (5.7)	8 (5.5)	3 (6.4)	7 (8.0)	6 (8.5)	1 (1.9)
Hb < 11 g/dL at baseline	20 (9.4)	12 (6.2)	9 (6.2)	3 (6.4)	16 (18.2)	2 (2.8)	2 (3.8)
Spontaneous non-ICH bleeding in the past 6 months or at any time, if recurrent *	10 (4.7)	5 (2.6)	5 (3.4)	0 (0)	8 (9.1)	1 (1.4)	1 (1.9)
Platelet count < 100 × 10^9^/L at baseline	2 (0.9)	1 (0.5)	1 (0.7)	0 (0)	2 (2.3)	0 (0)	0 (0)
Chronic bleeding diathesis	2 (0.9)	2 (1.0)	2 (1.4)	0 (0)	2 (2.3)	0 (0)	0 (0)
Liver cirrhosis with portal hypertension	0 (0)	0 (0)	0 (0)	0 (0)	0 (0)	0 (0)	0 (0)
Active malignancy within the past 12 months (excluding nonmelanoma skin cancer) §	5 (2.4)	3 (1.6)	3 (2.1)	0 (0)	5 (5.7)	0 (0)	0 (0)
ICH/stroke major †	2 (0.9)	1 (0.5)	0 (0)	1 (2.1)	2 (2.3)	0 (0)	0 (0)
Nondeferrable major surgery on DAPT	0 (0)	0 (0)	0 (0)	0 (0)	0 (0)	0 (0)	0 (0)
Recent major surgery or major trauma within 30 days before hospitalization	1 (0.5)	1 (0.5)	1 (0.7)	0 (0)	0 (0)	0 (0)	1 (1.9)
Age ≥ 75	88 (41.5)	78 (40.4)	56 (38.4)	22 (46.8)			
Moderate CKD (eGFR 30–59.99 mL/min)	58 (27.4)	54 (28.0)	43 (29.5)	11 (23.4)	36 (40.9)	19 (26.8)	3 (5.7)
Hb 11–12.9 g/dL for men and 11–11.9 g/dL for women at baseline	38 (17.9)	36 (18.7)	27 (18.5)	9 (19.1)	20 (22.7)	16 (22.5)	2 (3.8)
Spontaneous non-ICH bleeding within the past 12 months not meeting the major criterion *	0 (0)	0 (0)	0 (0)	0 (0)	0 (0)	0 (0)	0 (0)
Long-term use of p.o. NSAIDs or steroids	18 (8.5)	14 (7.3)	12 (8.2)	2 (4.3)	11 (12.5)	4 (5.6)	3 (5.7)
Any ischemic stroke at any time not meeting the major criterion	19 (9.0)	19 (9.8)	12 (8.2)	7 (14.9)	12 (13.6)	7 (9.9)	0 (0)

* Requiring hospitalization or transfusion. § Active malignancy is defined as diagnosis within 12 months and/or ongoing requirement for treatment. † Previous spontaneous ICH (at any time), previous traumatic ICH within the past 12 months, ischemic stroke within the past 6 months. Values are *n* (%). OAC: oral anticoagulation (direct oral anticoagulant or vitamin K antagonist); CKD: chronic kidney disease; eGFR: estimated glomerular filtration rate (CKD-EPI formula); Hb: hemoglobin; ICH: intracranial hemorrhage; DAPT: dual antiplatelet therapy; NSAID: non-steroidal anti-inflammatory drug; NSTE–ACS: non-ST-segment elevation acute coronary syndrome (excluding Type 2 myocardial infarctions); NSTEMI: non-ST-segment elevation myocardial infarction (Type 1); UAP: unstable angina pectoris.

## Data Availability

All data are available upon reasonable request by contacting the corresponding author.
